# Supplementary material for: Comparative multiomics analysis of cell physiological state after culture in a basket bioreactor
Source: Sci Rep. 2022 Nov 23;12:20161. doi: 10.1038/s41598-022-24687-4 (PMC9686226; doi:10.1038/s41598-022-24687-4)

36827971:+@NW_023666044.1:36839267:36839365:+@NW_023666044.1:36844255:36844382:+@NW_02366[illegible]

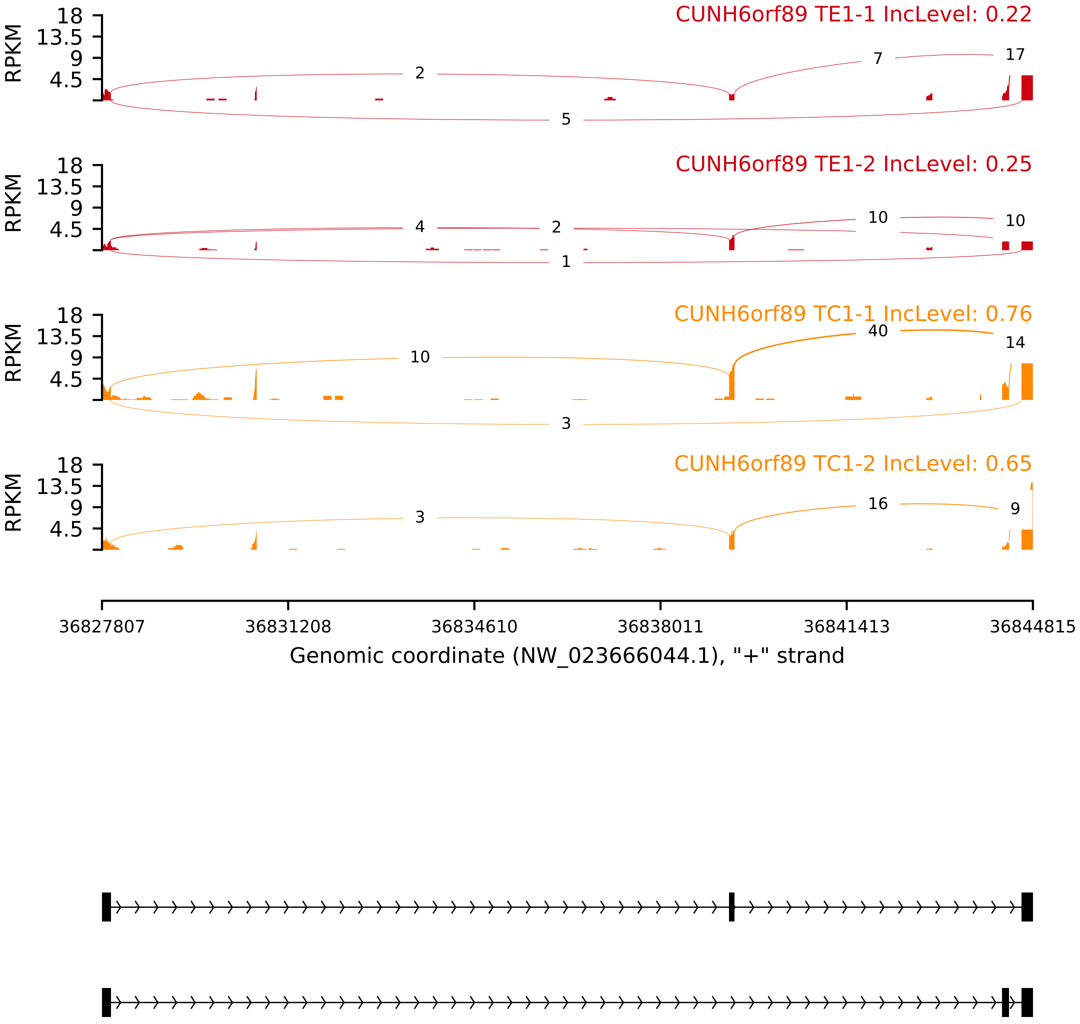

Supplement: Supplementary file 1 — Supplementary Information 1. [file 41598_2022_24687_MOESM1_ESM.zip › raw data/Transcriptomic raw data/7.AS/2.dasplot/TE1vsTC1/MXE/18_CUNH6orf89_NW_023666044.1_36827807_36827971_+@NW_023666044.1_36839267_36839365_+@NW_023666044.1_36844255_36844382_+@NW_023666044.1_36844609_36844816_+.pdf]
